# Liberty or life: the Aotearoa New Zealand perspective

**DOI:** 10.1017/S1092852924000579

**Published:** 2025-01-02

**Authors:** Justin Barry-Walsh

**Affiliations:** Forensic Psychiatrist, Fixated Threat Assessment Centre New Zealand, Te Whatu Ora Aotearoa, Wellington, New Zealand

**Keywords:** Aotearoa New Zealand, homelessness, Mental Health Act, prison, schizophrenia, fixated threat

## Abstract

A description is provided of the current situation in Aotearoa New Zealand with regard to compulsory treatment of people with schizophrenia. This is placed within the context of homelessness in New Zealand and the provision of services to the incarcerated mentally ill. There are high rates of homelessness and incarceration and services are struggling to meet their needs. This is particularly a problem for the indigenous population. The current Mental Health Act allows for compulsory treatment of people who as a result of schizophrenia are seriously impaired in their capacity to care for themselves, and this will include people where there is a nexus between homelessness and their illness. The Mental Health Act is being reformed, with a new act likely to emphasize autonomy and capacity to a greater degree. Finally, the author considers the learnings from 5 years working within the Fixated Threat Assessment Centre, which provides a unique perspective on these issues.

## Introduction

### Homelessness, prisons and mental illness

Homelessness is a major problem in Aotearoa New Zealand. According to the OECD report on homelessness, New Zealand’s rate of 2.17% of the population is the highest of any of the countries included in the survey.^1^ This can be partly explained by the broad definition of homelessness used in New Zealand (New Zealand includes those in emergency accommodation, homeless shelters, nonconventional accommodation such as mobile homes, those temporarily staying with family or friends, and those living in dilapidated buildings such as those without power and/or water) but does not fully account for the high rate in New Zealand. There are differences between ethnicities and Maori are overrepresented in the homeless population.^2^ A recent study in Auckland, New Zealand’s biggest city, identified that nearly 43% of all those living rough (meaning living in streets or public spaces without a shelter that can be defined as living quarters) in Auckland were Indigenous.^3^ Maori make up about 17% of the total New Zealand population. The latest homelessness data is based on the 2018 Census and trends over time are not clear. Official figures between 2013 and 2019 would suggest that the rate of homelessness in New Zealand only increased by 2%.^1^

The drivers of homelessness are similar to other countries. They include increasing problems with housing affordability and lack of available social housing.^2^ There is little information about the proportion of people with mental illness who are homeless. A survey in 2001 identified that 70% of all people with mental health problems receiving treatment within the public system had issues with homelessness or housing and 4% were recognized as homeless.^4^ Anecdotal and media reports would indicate mentally ill people struggle to be discharged from hospital because of a lack of available housing and impose an increased burden on those working with the homeless.^5^ Whether in New Zealand the rates of psychosis amongst homeless are elevated is unknown, but clinical experience and research from overseas would suggest that probably around 15 to 20% of the homeless have a psychotic illness,^6^ and it is thought this rate will be higher amongst those who are living rough.

New Zealand has by international comparison a high rate of incarceration. In 2018 there were 201/100000 population people in prison. There was growth in the prison population of 70% between 1997 and 2011 despite a decrease in the number of people being charged with criminal offenses. As of 31 March 2024, the prison population stood at 9508 of which 6.5% were women. This represents a rate of 179/100000. This rate is currently creeping up but had dropped in 2018 due to the changes in policy introduced by the previous government. Of further concern is the rise in the percentage of the prison population on remand. Between 2012 and 2020 the remand population increased by nearly 50%. Indigenous people are grossly over-represented in the prison population, in March 2024 52.4% identified as Maori.^7^

It is well established there are high rates of serious mental illness in the New Zealand prison population. A thorough survey in 1999 identified that 6% of prisoners at that time had schizophrenia or related psychotic illness^8^ In 2015 using a similar methodology 12% of people in prison were reporting psychotic symptoms.^9^

## Services

Skipworth et al examined whether the shift in models of care for people with mental illness was impacting the number of mentally ill people being incarcerated.^10^ This study explored whether the risk of imprisonment following discharge from the mental health unit had increased over recent years. It is important to note that there has been a marked decline in the number of inpatient beds available for the treatment of mental illness in New Zealand. In the last 20 years, there was a decline from around 50 per hundred thousand to 28 per hundred thousand.^10^ The average length of stay had fallen to 18 days and occupancy is often well over hundred percent. This is consistent with trends seen overseas.^10^ Skipworth et al found an increase in the number of people seen in prison services within 28 days of discharge from .6 to .9% of all discharges between 2012 and 2019. Maori were over-represented as were Pacific peoples. They identified those released from inpatient units where homelessness and lack of meaningful employment were issues were more likely to find themselves incarcerated. This was noted by them to be congruent with the recognized increase in homelessness and social deprivation that occurred in New Zealand over that time.

The provision of care for people with serious mental illnesses in prisons in New Zealand is the responsibility of regional forensic psychiatric services. There are five regional services in New Zealand.^11^ These services were developed in the early 1990s following a scathing review following several high-profile incidents in the community and prison.^12^ The model of care developed was influenced by the medium secure care model in the United Kingdom.

The Mental Health (Compulsory Assessment and Treatment) Act 1992^13^ allows for the transfer of acutely unwell prisoners to hospitals to secure hospitals for treatment and this is a major function of the regional inpatient units. However, they are also responsible for the treatment and rehabilitation of mentally disordered offenders coming through the Courts. The combination of necessarily long-term treatment of mentally disordered offenders found unfit to stand trial or not guilty by reason of insanity, in conjunction with the lack of proportionate growth in acute inpatient beds in forensic services to match the growth in prison population, has led to increasing pressure on forensic inpatient units. This is reflected in the rate of transfer from prison to hospital. In 2001, when the forensic services were relatively new and well-resourced, 2.31% of the overall prison population was transferred to the forensic units. By 2008 there had been a fall to 1.4%, and by 2015 these transfers were down to 0.67%.^14^ Consistent with this the ratio of prison muster to forensic inpatient beds was 28.11 in 2013. By 2019 this had risen to 39.47.^7^

This parlous state of affairs for the treatment of mental illness, including psychosis, in prison has been pointed out. In 2020 the directors of the forensic psychiatric services wrote a critical article in which they highlighted the ways in which the treatment of mental illness in prisons was failing, including failing to meet the standards required of New Zealand, as a signatory to international human rights agreements.^15^ This issue has recently been subject to media attention.^16^ The lack of appropriately trained forensic psychiatrists has also been noted.^17^ Of relevance, the Australian and New Zealand College of Psychiatrists are opposed to the use of involuntary treatment in prison, with good reason.^18^ In New Zealand, there is no scope for involuntary treatment of mental illness within prison.^14^

### Compulsory treatment in New Zealand

Compulsory treatment in New Zealand is permitted under the aegis of the Mental Health (Compulsory Assessment and Treatment) Act 1992.^13^ This act defines mental disorders and allows for a person to be detained and treated without consent if they are mentally disordered. Mentally disordered is defined as *“an abnormal state of mind (whether of a continuous or an intermittent nature), characterized by delusions, or by disorders of mood or perception, volition or cognition, of such a degree that it- (a) poses a serious danger to the health and safety of that person or others; or (b) seriously diminishes the capacity of that person to take care of himself or herself.”* People are initially assessed in a two-step process facilitated by what is known as a Duly Authorized Officer and, with an assessment by a doctor and a further assessment by a psychiatrist, may then be subject to compulsory assessment and treatment for 5 days, usually as an inpatient though occasionally this is done in the community. Within those 5 days, a decision has to be made as to whether they should be subject to further compulsory assessment and treatment for a period of 14 days after which time an application can be made to the Family Court for a compulsory treatment order. These orders can either be as an inpatient or for treatment in the community.^19^ Initially, orders can be made for a maximum of 6 months. A person made subject to compulsory treatment has the right to appeal both to a family court judge and subsequently to a tribunal. Repeat orders can be made for a further 6 months and, after recent reform, for a maximum of 1 year.

Several aspects of the definition of mental disorder merit further examination. The intent of the term mental disorder is deliberately to avoid reliance on diagnostic criteria.^20^ There are specific exclusions that include substance abuse, intellectual disability, offending behavior, sexual preference, or political, religious, or cultural beliefs.

For practical purposes, the terms delusion and disorder of mood and perception are relatively straightforward. However, cognition and volition have both been recognized as being more difficult to conceptualize and define, noting the lack of a generally accepted psychiatric meaning. It is thought for the most part, these criteria are utilized for identifiable serious mental illnesses such as psychosis or mood disorder.^20^ Personality disorders are neither specifically included nor excluded from the Mental Health Act.

Serious danger is generally understood to reflect the risk an individual may be violent toward others or alternatively to be at risk to themselves primarily through deliberate self-harm and attempted suicide. However, the concept can extend to other harms, such as the psychological harm caused by stalking behavior arising out of mental disorders.^19^

Of relevance to the issue of homelessness, seriously diminished capacity to care for oneself is *“not limited to the basic necessities of survival… It also includes “the multiplicity of other needs such as achieving financial security, maintaining proper social relationships, **maintaining stable accommodation**…”]* ^19^ On this basis, an individual who suffers from schizophrenia, and as a result of the combination of impairments arising from that has become homeless, may meet the criteria for mental disorder within the meaning of the Act. A case-by-case approach is recognized as being important in analyzing whether they meet the criteria.

The current Act in New Zealand, therefore, allows for the use of compulsory treatment in situations where, as a result of psychotic illness, there is serious impairment in a person’s capacity to care for themselves including rendering them homeless. Two questions arise from this. The first is how effective is this as an approach to the treatment of such individuals and the second is how often and what services may be available to assist such an individual?

Unsurprisingly, there is little limited information with regard to these questions. The issue as to whether compulsory treatment in the community is effective and ethical has been subject to debate. Beaglehole, Newton-Howes, and Frampton in 2021 commented on the intrusiveness of compulsory treatment orders, which in New Zealand means, for the most part, individuals are placed on depot antipsychotic medication and are subject to considerable intrusion as treating clinicians are able to enter premises and administer medication.^21^ If they refuse, they may be admitted to the hospital for the injection. The authors note a cultural aspect to this issue. In New Zealand there are higher rates of mental illness in Maori compared to non-Maori^22^ and Maori are more likely to be subject to compulsory inpatient and community treatment orders.^23^ There is also variation in the frequency of the use of compulsory treatment orders between jurisdictions in New Zealand.^23^ Encouragingly, Beaglehole et al found evidence that, for people with psychotic disorders, the use of compulsory treatment orders in the community both reduced the number of admissions they had by a factor of 18% and reduced their length of stay in hospital by about 3 days.^21^ This was utilizing a cohort design, and this made these findings more impressive as it may be the study design limited the ability to identify treatment effects for this group, as people who are more seriously unwell are more likely to be subject to compulsory treatment.

Although there is variability between services within New Zealand, often regions have assertive treatment teams to deal with those people with mental illness who are homeless and/or lacking in family support. These services often work with other agencies both publicly funded and NGOs who provide support and often accommodation for such individuals.

### Reform of the Mental Health Act

In 2018 the government commissioned a review of mental health and addiction in New Zealand known as He Ara Oranga or the Government Enquiry into Mental Health and Addiction.^24^ This wide-ranging review was released in November 2018 after extensive community engagement. It identified major failings in the current mental health services. It recommended a shift from “big psychiatry” to “big community” which was conceptualized as being a more holistic, recovery-based, culturally sensitive approach to dealing with people suffering from distress (rather than mental illness) and targeting around 20% of the population instead of the 3% that had been targeted previously in the development of mental health services. It noted *“People called for repeal and replacement of the mental health (Compulsory Assessment and Treatment) Act 1992… And an end to seclusion and restraint… The Mental Health Act embeds archaic and risk-averse attitudes that cause clinicians to opt too readily for coercion and control.”*

He Ara Oranga when released was not without controversy. It was noted the language within He Ara Oranga had a lineage that dated back to the reforms from the mid-20th century onwards, and that saw the deinstitutionalization and closure of psychiatric hospitals which have subsequently led to low numbers of hospital inpatient psychiatric beds in the English-speaking world. It was observed that ironically New Zealand already had “small psychiatry” by international standards. As a result, concerns have been raised about whether reform to mental health services and the Mental Health Act as recommended will improve the mental health of New Zealanders.^25^

The government on 6 May 2019 decided to repeal the Act. A high-level discussion document was released in 2021^26^ and cabinet papers released in August 2023^27^ provided clues as to the form the new Act is likely to take. It will focus on recovery, advanced directives, the inclusion of family and cultural bodies and incorporate concepts from Te Tiriti O Waitangi (The Treaty of Waitangi, a foundational document signed in 1840 between representatives of the British Crown and some Maori iwi (tribes.)) It explicitly intends to reduce and ultimately end seclusion and reduce restraint. The intention is to minimize compulsion. There is an emphasis within the materials released thus far on the preservation of autonomy. It is apparent that there will be a shift to a three-limb test for mental disorder—an abnormal state of mind, serious danger or diminished capacity to care, and a requirement for incapacity. The details of this remain unclear, but an update from the Ministry of Health in April 2024, in the context of a round of consultation with stakeholders, confirms the direction the Act will take as part of an overhaul of services.^28^ It is planned to emphasize the preservation of autonomy with supported decision-making, greater incorporation of cultural and family input, reduction in restrictive practices, and strengthened rights.

The move away from the use of language such as mental illness to well-being and distress highlights a key issue. The reforms and approaches outlined within He Ara Oranga and followed in the plans to repeal the Act may work well for individuals in distress where medicalization of their distress is unhelpful or harmful. However, there is a real possibility that such an approach will not meet the needs of individuals with recognizable serious mental illnesses and associated problems with aggression and violence.^10^ Based on what is known at this stage it seems likely that it will be harder to use compulsory treatment, both due to the added requirement for impairment of capacity and because of the expressed intent and changes described above.

### A fixated threat perspective

Half of my time is spent working at the Fixated Threat Assessment Centre New Zealand (FTACNZ.) This is based on the United Kingdom (UKFTAC) and Australian models and sees police and mental health staff working together to deal with individuals who correspond or approach in concerning and threatening ways with Members of Parliament. The cornerstone of this service is research that identifies these people are commonly fixated and with high rates of serious mental illness.^29^ Since its inception, initially as a small consultative group, and since July 2019 as a full-time service, FTACNZ has dealt with over 700 referrals. Half of these those referred are readily identified as suffering from a psychotic illness with another 10% suffering from a mood disorder, usually bipolar affective disorder. Often, these individuals have either fallen out of treatment or have never been engaged in treatment. As senior clinical staff from UKFTAC have reflected, working in these services provides an opportunity to observe and reflect on the vicissitudes and effectiveness of services for those with serious mental illnesses, usually psychosis.^30^ I find myself in a similar position in New Zealand. Commonly those referred have serious psychotic illnesses. Often their communications are chaotic, they may be petitioning or pleading for help with delusion-based persecution. At times they are threatening, and their behavior can be both concerning and bizarre to recipients including staff of electorate offices scattered around the country and those who work at Parliament in Wellington. Usually, the distress these people are experiencing is palpable in their communication.

Commonly these individuals are itinerant and therefore homeless. Frequently, they are disorganized in their social circumstances, strongly motivated by their beliefs, and may travel from various parts of the country to Wellington where they may present at embassies and parliament before vanishing into the streets again. Sometimes they live in cars. These people pose several practical and ethical problems. Often, while their behaviors may be disorganized and bizarre, and they appear to be homeless, we lack sufficient information to be satisfied they meet the legal threshold for compulsory treatment. Further, the logistics of accessing such treatment becomes complex. They may present after hours, in which case it may fall to the police to firstly recognize they are mentally ill and secondly that their detention is justified (if they have committed a criminal offense they can be arrested, and if they are mentally ill in public, under section 109 of the mental health (Compulsory Assessment and Treatment) Act 1992.^13^ they can be held by the police pending assessment.) A further obstacle to treatment is whether police can elicit an appropriate response from mental health services.

Sometimes, these logistical problems are resolved when the person commits a usually relatively minor offense, often understandable in the context of their illness, and then is forced to attend court where they may be assessed, through the Court Liaison arm of Forensic Mental Health Services. Often, however, we may be aware of individuals who we believe are seriously mentally ill, and likely seriously impaired in their capacity to care for themselves, as well as distressed and causing harm to themselves and those around them yet we are unable to locate them or arrange for them to have assessment and provision of treatment they may need. Often, as psychosis and behavior ebb and flow, the individual may cease to communicate. However, occasionally and tragically, they may engage in low-base rate but high-harm events. In this regard, the recent stabbing and death of six individuals at the Bondi Junction shopping center^31^ forces us to consider how we should approach such individuals and whether there is a better way of dealing with the chaos and distress caused by their illness. It would be rare for such individual to act in such an appallingly violent and tragic manner, yet equally, their combination of distress, psychosis, and poor engagement socially and with mental health services surely merits a more robust, targeted, and compassionate approach.

## Conclusions

Existing Mental Health Act legislation in New Zealand does allow for compulsory treatment of people with schizophrenia who, as a result of their illness, are seriously incapacitated in their ability to care for themselves, with a potential outcome of homelessness. Throughout the country, there are services geared toward treating these individuals, although the extent to which they can meet their needs and their effectiveness overall is unclear. Services within prisons have degraded since their inception in the early 1990s because of the increase in demand and the lack of provision of further inpatient beds and services to meet the demand. Similarly, adult mental health services increasingly struggle as the number of beds available to admit seriously mentally ill individuals has decreased. Reform of the current Mental Health Act is likely to be in the direction of emphasizing autonomy, minimizing the use of compulsory treatment, and likely making it more difficult to coercively treat homeless individuals with psychosis. The FTAC perspective allows for a qualitative, meta-position that illustrates the complexities of dealing with homeless people with schizophrenia, endurance of suffering for these people, and the harm they can cause others.
